# Estimation of brain tissue response by electrical stimulation in a subject-specific model implemented by conductivity tensor imaging

**DOI:** 10.3389/fnins.2023.1197452

**Published:** 2023-05-23

**Authors:** Nitish Katoch, Youngsung Kim, Bup Kyung Choi, Sang Woo Ha, Tae Hoon Kim, Eun Ju Yoon, Sang Gook Song, Jin Woong Kim, Hyung Joong Kim

**Affiliations:** ^1^Department of Biomedical Engineering, Kyung Hee University, Seoul, Republic of Korea; ^2^Office of Strategic R&D Planning (MOTIE), Seoul, Republic of Korea; ^3^Department of Neurosurgery, Chosun University Hospital and Chosun University College of Medicine, Gwangju, Republic of Korea; ^4^Medical Convergence Research Center, Wonkwang University Hospital, Iksan, Republic of Korea; ^5^Department of Radiology, Chosun University Hospital and Chosun University College of Medicine, Gwangju, Republic of Korea

**Keywords:** electrical stimulation, conductivity tensor, transcranial direct current stimulation, magnetic resonance imaging, electric field, current density

## Abstract

Electrical stimulation such as transcranial direct current stimulation (tDCS) is widely used to treat neuropsychiatric diseases and neurological disorders. Computational modeling is an important approach to understand the mechanisms underlying tDCS and optimize treatment planning. When applying computational modeling to treatment planning, uncertainties exist due to insufficient conductivity information inside the brain. In this feasibility study, we performed *in vivo* MR-based conductivity tensor imaging (CTI) experiments on the entire brain to precisely estimate the tissue response to the electrical stimulation. A recent CTI method was applied to obtain low-frequency conductivity tensor images. Subject-specific three-dimensional finite element models (FEMs) of the head were implemented by segmenting anatomical MR images and integrating a conductivity tensor distribution. The electric field and current density of brain tissues following electrical stimulation were calculated using a conductivity tensor-based model and compared to results using an isotropic conductivity model from literature values. The current density by the conductivity tensor was different from the isotropic conductivity model, with an average relative difference |*rD*| of 52 to 73%, respectively, across two normal volunteers. When applied to two tDCS electrode montages of C3-FP2 and F4-F3, the current density showed a focused distribution with high signal intensity which is consistent with the current flowing from the anode to the cathode electrodes through the white matter. The gray matter tended to carry larger amounts of current densities regardless of directional information. We suggest this CTI-based subject-specific model can provide detailed information on tissue responses for personalized tDCS treatment planning.

## 1. Introduction

Electrical stimulation of brain has been used for the treatment of neuropsychiatric and neurodegenerative diseases (Stagg and Nitsche, 2011). Transcranial direct current stimulation (tDCS) is a typical neuro-stimulation technique that delivers low-intensity direct current (DC) into the brain through a pair, or through multiple surface electrodes. The injected current may cause exogenous modulation of neuronal membrane potentials, leading to an enhancement of brain functions (Nitsche and Paulus, 2000; Woods et al., 2016). The tDCS is clinically used for enhancing motor or memory functions and treatments of neuropsychiatric and neurodegenerative diseases (Fregni et al., 2006; Márquez-Ruiz et al., 2012; Reinhart et al., 2015; Lindenmayer et al., 2019). When electrical stimulation is applied to the head, the injected current through the brain generates voltage, electric field, and current density, which are affected by head geometry, electrode configuration, and the internal conductivity distribution (Bikson et al., 2012; Woods et al., 2016). To calculate these internal distributions, modeling studies have focused on designing electrode montages, modifying the sizes and shapes, and adjusting the amount of current applied (Miranda et al., 2006; Bikson et al., 2012). Moreover, subject-specific head models have been developed by segmenting structural MR images to handle the effects of the head geometry and electrode configuration (Miranda et al., 2006; Bikson et al., 2012; Datta et al., 2012). Several studies have proposed methods to incorporate *in vivo* distribution of internal conductivity and/or its tensors in individual subjects (Rampersad et al., 2014; Shahid et al., 2014; Opitz et al., 2015). However, its application was limited due to the lack of proper ways to measure subject-specific conductivity distribution at low-frequency ranges.

A comprehensive computational approach has been proposed to optimize the design of tDCS for clinical use (Bikson et al., 2012; Lee et al., 2021). Although the idea of subject-specific brain conductivity recognizes as an important factor (Puonti et al., 2020), the use of individual anisotropic conductivity has not been widely applied in modeling studies (Lee et al., 2021). Meanwhile, Tuch et al. introduced a linear model based on the physical relationship between conductivity tensor and water diffusion tensor (Tuch et al., 2001). Based on the idea of a cross-property relationship, several conductivity tensor models were developed and utilized for modeling of brain stimulation (Rampersad et al., 2014; Shahid et al., 2014). Recently, Katoch et al. reported that water diffusion tensor models may not fully address the effect of ion concentrations on conductivity distribution, therefore, novel model using conductivity tensor was suggested to precisely estimate the anisotropic distribution of brain (Katoch et al., 2021).

Diffusion-tensor magnetic resonance electrical impedance tomography (DT-MREIT) is currently used to image brain responses during electrical stimulation (Kwon et al., 2014; Jeong et al., 2016). The basis of DT-MREIT is that the water diffusion tensors have the same directional property as conductivity tensors (Tuch et al., 2001). There have been several experimental studies of DT-MREIT in phantoms, animals, and human subjects (Kwon et al., 2014; Jeong et al., 2016; Chauhan et al., 2018), but clinical uses may be limited due to the requirement for imaging currents of a few milliamperes through multiple surface electrodes during MRI scans. The conductivity tensor imaging (CTI) method, which does not require external current injection, was developed recently (Sajib et al., 2018). The CTI consists of a combination of magnetic resonance electrical properties tomography (MREPT) and multi-b-value diffusion weighted imaging (DWI). To reconstruct the conductivity tensor, MREPT is used to obtain high-frequency conductivity (at the Larmor frequency), and multi-b-value DWI is used to separate the information on both the extracellular and intracellular spaces. This method was validated in the designed phantoms and animal imaging studies using a 3 T MRI scanner and its feasibility was evaluated (Sajib et al., 2018). Specifically, Katoch et al. developed a model that mimicked a cell structure with giant vesicles and separated extracellular and intracellular spaces using a CTI method (Katoch et al., 2018). The relative errors between the conductivity tensor images and *in vitro* measurements of the giant vesicle suspensions were found to be about 1.1 to 11% (Katoch et al., 2018; Choi et al., 2020). In addition, Choi et al. reported that CTI can distinguish the contrast between ion concentrations and their mobility (Choi et al., 2020, 2023). The advantage of CTI is that it is easily implemented in a clinical MRI scanner without addition of hardware components. *In vivo* CTI imaging of the human brain was reported, and resulting conductivity values of white matter, gray matter, and cerebrospinal fluid were compared with those previously reported in the literature (Katoch et al., 2018; Jahng et al., 2021). Therefore, CTI is the latest method to image tensor information along with conductivity distributions inside the brain, without the need to inject imaging currents.

In this feasibility study, we performed *in vivo* CTI experiments of entire human brains and implemented subject-specific conductivity tensor-based head models to evaluate the brain response to electrical stimulation. We acquired MR images for both structural and CTI information from two normal volunteers using a 3 T clinical MRI. After segmenting the structural MR images of the heads, three-dimensional finite element models were constructed. A subject-specific head model was implemented by incorporating the conductivity tensor images into the geometrical finite element model. Applying electrical stimulation to electrode montages of tDCS, the electric field and current density of the brain were calculated using a conductivity tensor-based head model and its performance evaluated by comparing it to the isotropic conductivity models as a tool for personalized tDCS treatment planning.

## 2. Subjects and methods

All experimental procedures were approved by the institutional review board of Kyung Hee University (KHSIRB-18-073) and carried out in accordance with the relevant guidelines and regulations. Two healthy male volunteers (26 and 28 years old) participated and informed consent was obtained from the volunteers before the imaging experiments.

### 2.1. Imaging experiments

**Figure 1:**
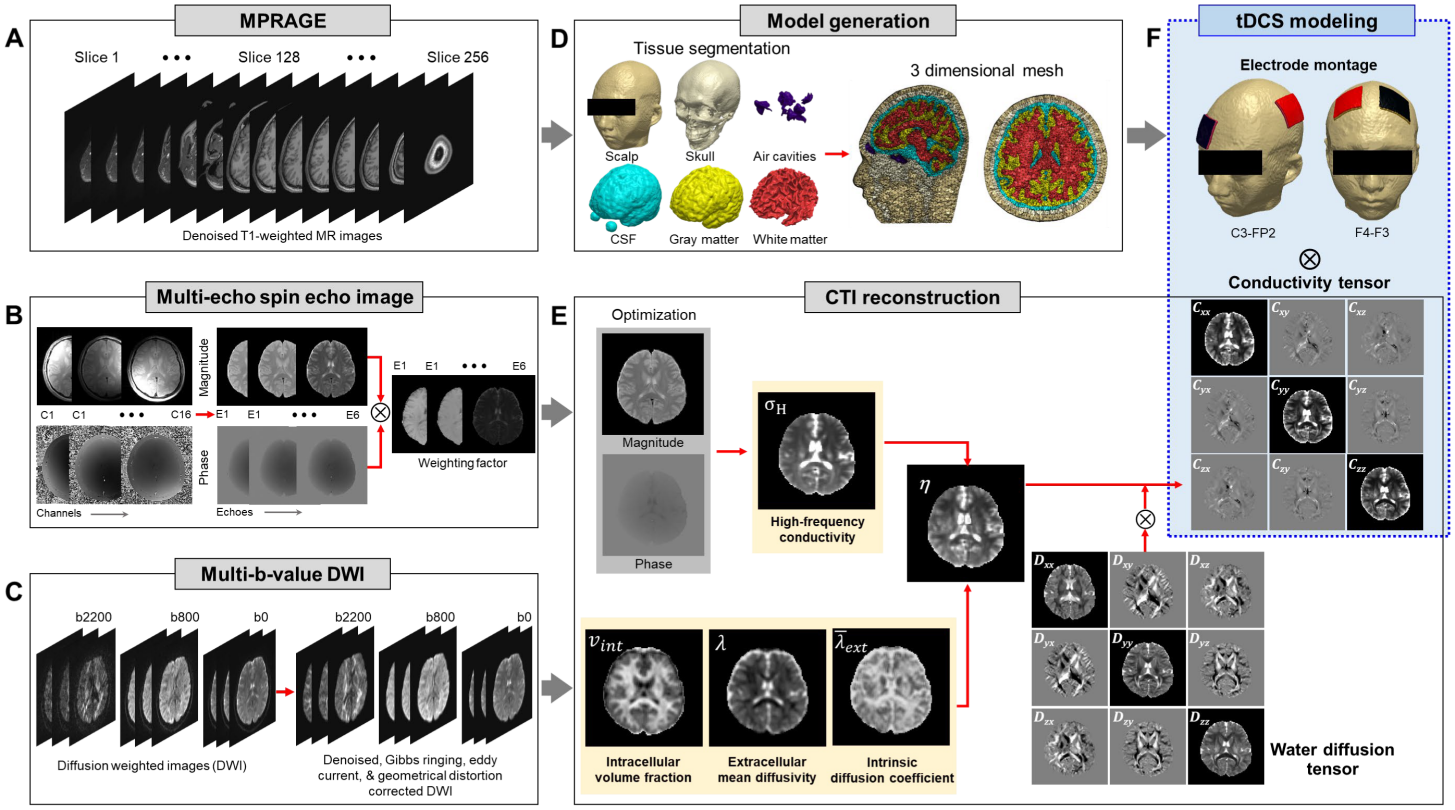
Schematic illustration of the conductivity tensor-based head model. High-resolution anatomical MR images **(A)** are used to generate the model. Multi-echo spin-echo images **(B)** and multi-*b*-value diffusion weighted images **(C)** are used to reconstruct the conductivity tensor of brain. Three-dimensional head model **(D)** and conductivity tensor information of the brain **(E)** are combined to estimate the response to tDCS electrode montages **(F)**.

The imaging experiments were performed on a 3 T MRI scanner (Magnetom Skyra, Siemens Healthcare, Erlangen, Germany) equipped with a 16-channel head coil. For structural images of the head (Figure 1A), T1-weighted magnetization prepared rapid gradient echo (MPRAGE) sequence was applied with a cubic voxel of 1 mm edge length in sagittal plane. The parameters were as follows: repetition time (TR)/echo time (TE) = 1200/1.79 ms; flip angle = 10°; bandwidth = 510 Hz/Px; field of view (FOV) = 260 × 260 mm^2^; slice per slab = 192; slice thickness = 1 mm (no gap). The acquisition time was 5 min and 4 s. CTI requires two separate MR scans shown in Figures 1B,C. For high-frequency conductivity images by MREPT method (Figure 1B), the multi-echo spin-echo (MSE) pulse sequence with multiple refocusing pulses was adopted to acquire B1 phase maps. The parameters were as follows: TR/TE = 1500/15 ms; flip angle = 90°; bandwidth = 250 Hz/Px; number of echoes = 6; number of slices = 30; slice thickness = 4 mm; matrix size = 128 × 128 and FOV = 260 × 260 mm^2^. The voxel size was 2 × 2 × 4 mm^3^. The acquisition time for obtaining high-frequency conductivity images covering the whole brain area was 19 min.

To separate the influences of the extracellular and intracellular spaces, multi-band echo-planar imaging (EPI) sequence was used to acquire two-shell DWI (Figure 1C). A multi-band factor of 3 was used with the three-band RF excitation and axial spin-echo EPI (SE-EPI) readout with phase encoding (PE) in the anterior–posterior (AP) direction. The diffusion gradient was applied in 30 and 64 directions with two *b*-values of 800 and 2,200 s/mm^2^. The parameters were as follows: TR/TE = 2000/80 ms; flip angle = 90°; number of slices = 30; slice thickness = 4 mm; matrix size = 128 × 128 and FOV = 260 × 260 mm^2^. The acquisition time was 9 min. An additional MR scan was obtained using a non-diffusion sensitizing gradient (*b* = 0 s/mm^2^) along the posterior–anterior phase encoding (PE) directions. The total acquisition time to implement the subject-specific model using CTI was 35 min.

### 2.2. Brain segmentation and model generation

High-resolution MPRAGE images were bias-corrected and preprocessed by applying a median filter to reduce noise (Smith et al., 2004). The automated segmentation algorithms in Statistical Parametric Mapping software (SPM 12, Wellcome Trust Centre for Neuroimaging, London, United Kingdom) were used to generate tissue probability maps. The head was segmented into six tissue types; scalp, skull, air cavity, eye, cerebrospinal fluid (CSF), gray matter (GM), and white matter (WM) (Figure 1D). After generating the initial tissue probability maps, a Matlab (Mathworks, Natick, United States) based algorithm (Huang et al., 2013) was used to correct tissue discontinuities and segmentation errors. The segmented tissue data was imported into ScanIP software (Simpleware, Synopsys, Exeter, United Kingdom) to build a three-dimensional head model (Figure 1D). The overlaps and unassigned pixels in the segmented data were removed by manual correction and all slices were confirmed to ensure proper tissue classifications. We used a recursive Gaussian filter with a kernel size of 1 × 1 × 1 to smooth the masks of tissue surfaces. Two sponge-pad electrodes with a size of 50 × 50 mm^2^, were attached on the model to stimulate the brain. The thickness of the sponge-pads was 3 mm and the electrode was 2 mm thickness. The finite element meshes of the head models include 1.5 million tetrahedral and 0.3 million triangular elements with an average element quality of 0.678.

### 2.3. Conductivity tensor image reconstruction

Conductivity tensor images were reconstructed using an MRCI toolbox which is available at http://iirc.khu.ac.kr/toolbox.html (Sajib et al., 2017). Acquired MR data (including anatomical images, B1 phase map, and DWI) was re-oriented to standard MNI space and preprocessed using the MRtrix3^1^ and FMRIB software library (FSL 6.03v, www.fmrib.ox.ac.uk/fsl) (Smith et al., 2004; Tournier et al., 2019). The B1 phase maps and DWI were registered with the anatomical T2-weighted images to minimize geometrical mismatches. The high-frequency conductivity images were reconstructed using a method proposed by Gurler and Ider (2017). The diffusion data were corrected for eddy-current effects and geometrical distortions by affine registration of the directional images of B0 using FSL software (www.fmrib.ox.ac.uk/fsl) with the FLIRT routine (Smith et al., 2004). The following CTI formula was used for all conductivity tensor image reconstructions (Katoch et al., 2018; Sajib et al., 2018):


(1) $$C=\frac{\left(1-v_{\mathrm{int}}\right)\sigma_{H}}{v_{\mathrm{int}}\beta \lambda +\left(1-v_{\mathrm{int}}\right){\bar{\lambda }}_{\mathrm{ext}}}D_{e}^{w}=\eta D_{e}^{w}$$

where **C** is the low-frequency conductivity tensor, *σ_H_* is the high-frequency conductivity at the Larmor frequency, $v_{\mathrm{int}}$ is the intracellular volume fraction, *β* is the ion concentration ratio of the intracellular and extracellular spaces, λ is the intrinsic diffusion coefficient, ${\bar{\lambda }}_{\mathrm{ext}}$ is the extracellular mean diffusivity, and **D**_e_^w^ is the extracellular water diffusion tensor (Figure 1E). The details of conductivity tensor reconstruction procedures followed the works of Jahng et al. (2021). Finally, the reconstructed conductivity tensor images were incorporated into the subject-specific head models.

### 2.4. Modeling of electrical stimulation

Two electrode montages were modeled to visualize the brain response during tDCS using a CTI head model (Figure 1F). The first montage consisted of an anode positioned in the C3 (motor cortex) and a cathode in the FP2 (supra-orbital). The second montage consisted of an anode in the F4 (right dorsolateral prefrontal cortex, DLPFC) and a cathode in the F3 (left DLPFC). The anode and cathode are denoted as *ε_A_* and *ε_C_*, respectively, and currents were injected from *ε_A_* to *ε_C_*. The cathode *ε_C_* was chosen as the voltage reference electrode for all numerical computations. When a DC current of *I* mA is injected between two electrodes *ε_A_* and *ε_C_*, the voltage *u* inside the head denoted as Ω with its boundary ∂Ω satisfies the following partial differential equation (Seo and Woo, 2012):


(2) $$\{\begin{array}{c} \nabla \cdot C\nabla u\left(r\right)=0\;in\;\Omega \\ -C\nabla u\left(r\right)\cdot n=g\left(r\right)\;on\;\partial \Omega \backslash \left(\varepsilon_{A}\cup \varepsilon_{C}\right) \end{array}$$

where **C** is the conductivity tensor, n is the outward unit normal vector on the boundary ∂Ω, and g is the Neumann boundary condition of the injected current. The voltage *u* (V), electric field −∇*u* (V/m), and current density − C∇*u* (A/m^2^) were numerically calculated using COMSOL Multiphysics (COMSOL, Burlington, United States) software. The conjugate gradient method with a relative tolerance of 1 × 10^−6^ was used to solve linear systems of equations. The amplitude of the injected current *I* was 2 mA and the average current density under the electrode was 0.8 A/cm^2^. The total computation time to obtain *u*, −∇*u*, and − C∇*u* was about 9 min.

### 2.5. Evaluation of CTI-based head model

The electric field and current density of the brains were calculated using a conductivity tensor-based head model and compared with those using the isotropic conductivity of brain tissues from literature values. For the conductivity tensor models, we used the reconstructed conductivity tensor images for the pixels belonging to the white matter, gray matter, and CSF regions. The isotropic low-frequency conductivity values from the literature were used for the conductivity of the other tissues such as scalp, skull, air cavity, and eye. Meanwhile, we used the isotropic literature values for all pixels for the isotropic conductivity model (Gabriel et al., 1996; Baumann et al., 1997; Datta et al., 2009; Bikson et al., 2012; Huang et al., 2013). The values (S/m) were as follows: air = 1 × 10^−15^, scalp = 0.47, skull = 0.01, eye = 2.00, CSF = 1.79, gray matter = 0.27, white matter = 0.14, electrode = 5.99 × 10^7^, and saline-soaked sponge = 1.00. For comparison of current densities between the two models, we analyzed the relative difference |*rD*| defined as follows:


(3) $$\mathrm{Relative difference}\left|rD\right|=\frac{\sum_{i=1}^{N}J_{\mathrm{CTI}}-J_{\mathrm{ISO}}}{\sum_{i=1}^{N}J_{\mathrm{CTI}}}\times 100\%$$

where J_CTI_ and J_ISO_ are the magnitude of current density at each pixel from the conductivity tensor and isotropic conductivity models, respectively. A high error indicates that there is a significant difference between two current densities.

## 3. Results

### 3.1. CTI of human brain

**Figure 2:**
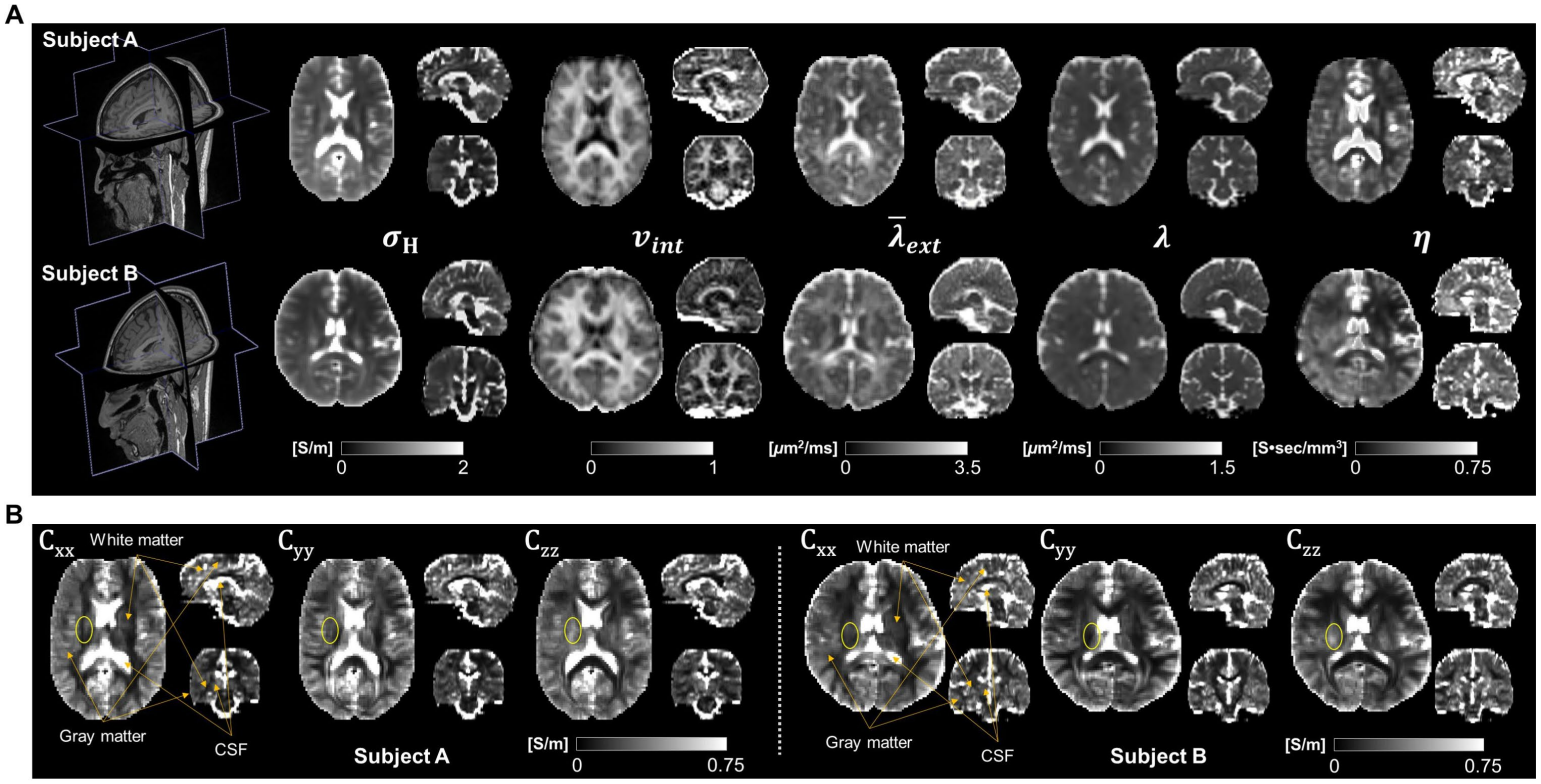
Typical conductivity tensor images acquired from two normal brains. High-frequency conductivity ( $\sigma_{H}$ ), intracellular volume fraction ( $v_{\mathrm{int}}$ ), extracellular mean diffusivity ( ${\bar{\lambda }}_{ext}$ ), intrinsic diffusion coefficient ( λ ) and scale factor ( η ) images **(A)** are used to calculate the conductivity tensors at three different fiber directions **(B)**.

Figure 2 shows the reconstructed conductivity tensor images of two normal brains using the CTI method. In Figure 2A, the high-frequency conductivity (*σ_H_*) and intermediate images (including intracellular volume fraction, $v_{\mathrm{int}}$ ; intrinsic and extracellular water diffusion coefficients, λ and ${\bar{\lambda }}_{ext}$ ; scale factor, *η*) were used to reconstruct low-frequency conductivity tensors shown in Figure 2B. To reconstruct the diffusion coefficients ( $v_{\mathrm{int}}$ , λ and ${\bar{\lambda }}_{ext}$ ) of brain we used the software available at https://ekaden.github.io (Kaden et al., 2016). The conductivity tensor images in the white matter, gray matter, and CSF regions were clearly distinguished (orange arrows) and showed different signal intensities in the white matter depending on the fiber direction (yellow circle) (Figure 2B).

### 3.2. Brain tissue response by electrical stimulation

**Figure 3:**
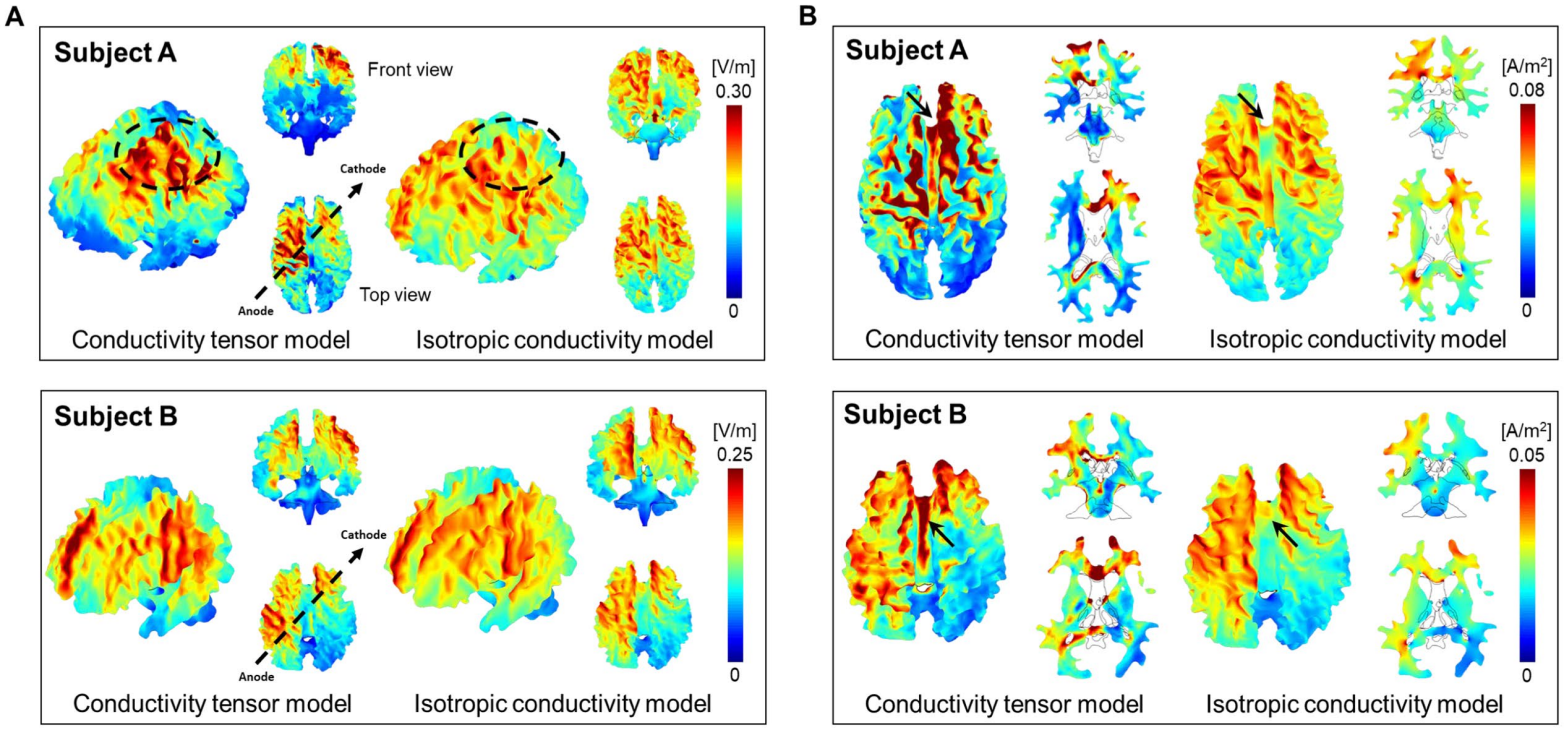
Brain response by the tDCS in the white matter of two normal brains. The three-dimensional images of electric fields **(A)** and current densities **(B)** were calculated from both the conductivity tensor model and isotropic conductivity tensor model.

Figure 3 shows the results of brain responses to tDCS focusing on the white matter. The electric field (Figure 3A) and current density (Figure 3B) distributions were obtained from both the CTI-based head model and the isotropic conductivity model with literature values. The electric field by the conductivity tensor model showed higher signal intensity in the activated area, but the activated area was wider in the isotropic conductivity model (Figure 3A). The current density in the conductivity tensor model showed a high signal intensity in the activated area, but the activated area was similar to the isotropic conductivity model (Figure 3B). The dynamic ranges of the electric field and current density were wider in subject A than in subject B. Table 1 summarizes the electric field and current density measurements in gray matter, white matter, and entire brain tissues. The conductivity tensor showed higher signal values than the isotropic conductivity model in all brain tissues. The averaged current density of the conductivity tensor in the corpus callosum (CC) were 0.073 A/m^2^ and 0.061 A/m^2^ in two subjects, respectively, which is twice as much as that in the isotropic conductivity model (0.034 A/m^2^ and 0.022 A/m^2^). This could also stem from the fact that conductivity of CC ranged 0.20 to 0.75 S/m, which is higher than that of 0.14 S/m in isotropic conductivity model.

**Table 1:** Measurements of the electric field and current density in gray matter, white matter, and entire brain tissues from isotropic and conductivity tensor models.

Model		Electric field [V/m]			Current density [A/m^2^]		
		Gray matter	White matter	Entire brain	Gray matter	White matter	Entire brain
Subject A	Conductivity tensor	0.104 ± 0.060	0.117 ± 0.063	0.116 ± 0.073	0.039 ± 0.026	0.032 ± 0.027	0.042 ± 0.032
	Isotropic conductivity	0.087 ± 0.035	0.112 ± 0.029	0.098 ± 0.057	0.023 ± 0.010	0.017 ± 0.005	0.042 ± 0.053
Subject B	Conductivity tensor	0.099 ± 0.042	0.118 ± 0.045	0.097 ± 0.049	0.033 ± 0.021	0.024 ± 0.011	0.043 ± 0.032
	Isotropic conductivity	0.076 ± 0.029	0.097 ± 0.031	0.074 ± 0.039	0.021 ± 0.008	0.014 ± 0.004	0.046 ± 0.048

### 3.3. Comparison of brain tissue response between two models

**Figure 4:**
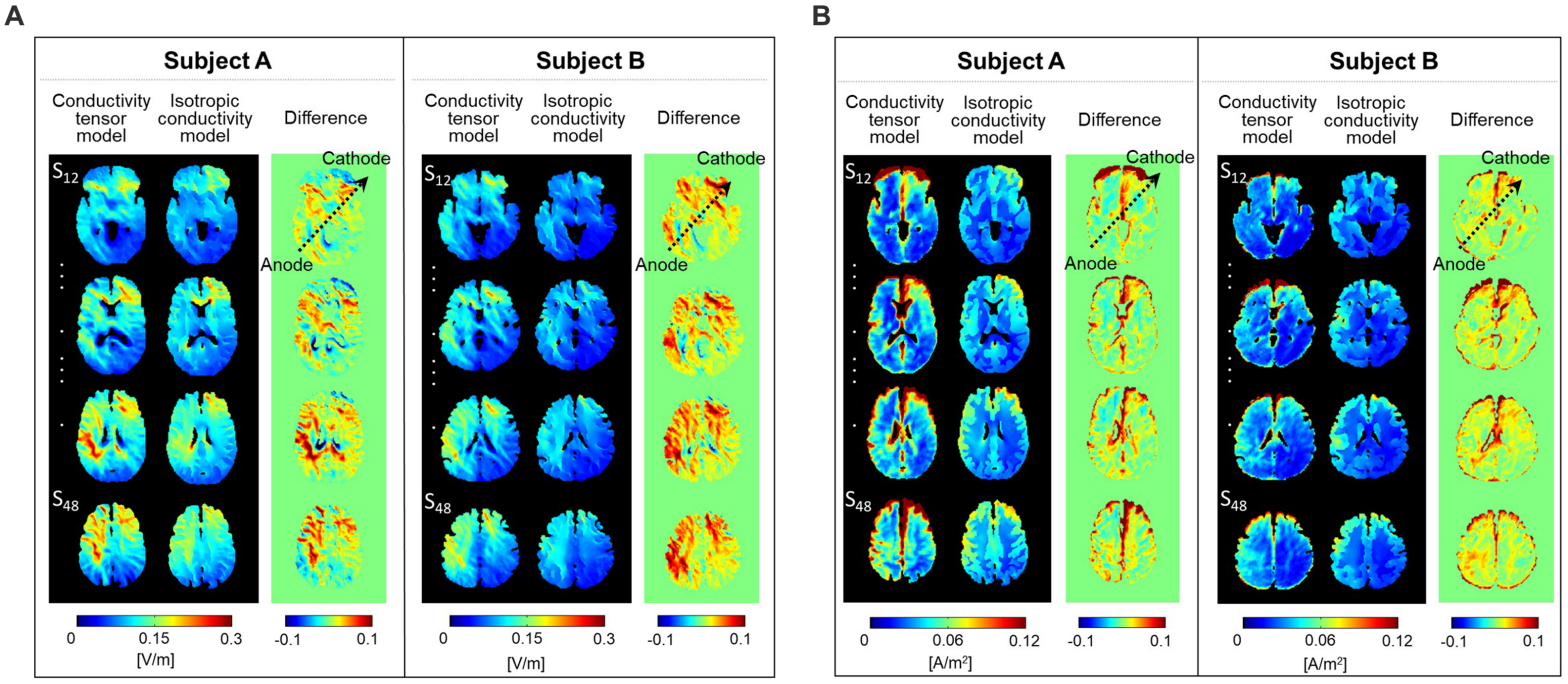
Difference images between the conductivity tensor model and isotropic conductivity model. The electric field **(A)** and current density **(B)** were calculated at a single slice whose position was chosen at 12 mm from the skull.

Figure 4 shows the difference images between the two models. From difference images of the electric field (Figure 4A), the conductivity tensor model showed high signal intensity around the electrode position. The current density showed high signal intensity along the direction of current flow between the two electrodes (black arrows) (Figure 4B). The signal intensity from model comparisons was different between the two subjects in both the electric field and current density.

**Figure 5:**
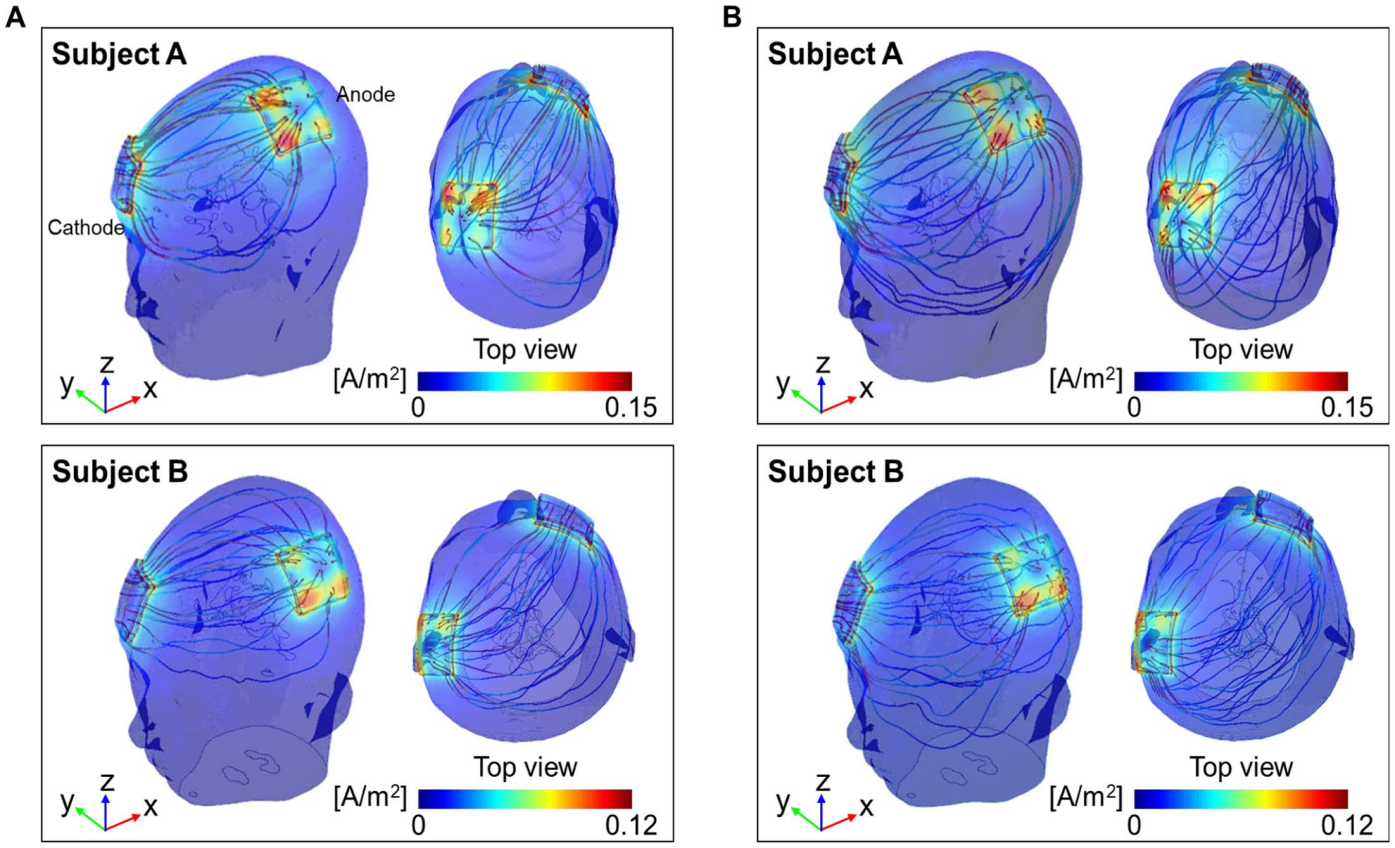
Distribution of current density streamlines inside the two brains. The colored streamline represents the magnitude of the current density obtained from the conductivity tensor **(A)** and the isotropic conductivity **(B)** models.

Figure 5 shows the current density streamlines from the anode to the cathode of the two models. The streamlines in the conductivity tensor model showed a concise and concentrated distribution between the electrodes (Figure 5A), while the isotropic conductivity model showed a wider distribution (Figure 5B). The dynamic range of the current density streamline was slightly wider in subject A than in subject B for both models.

**Figure 6:**
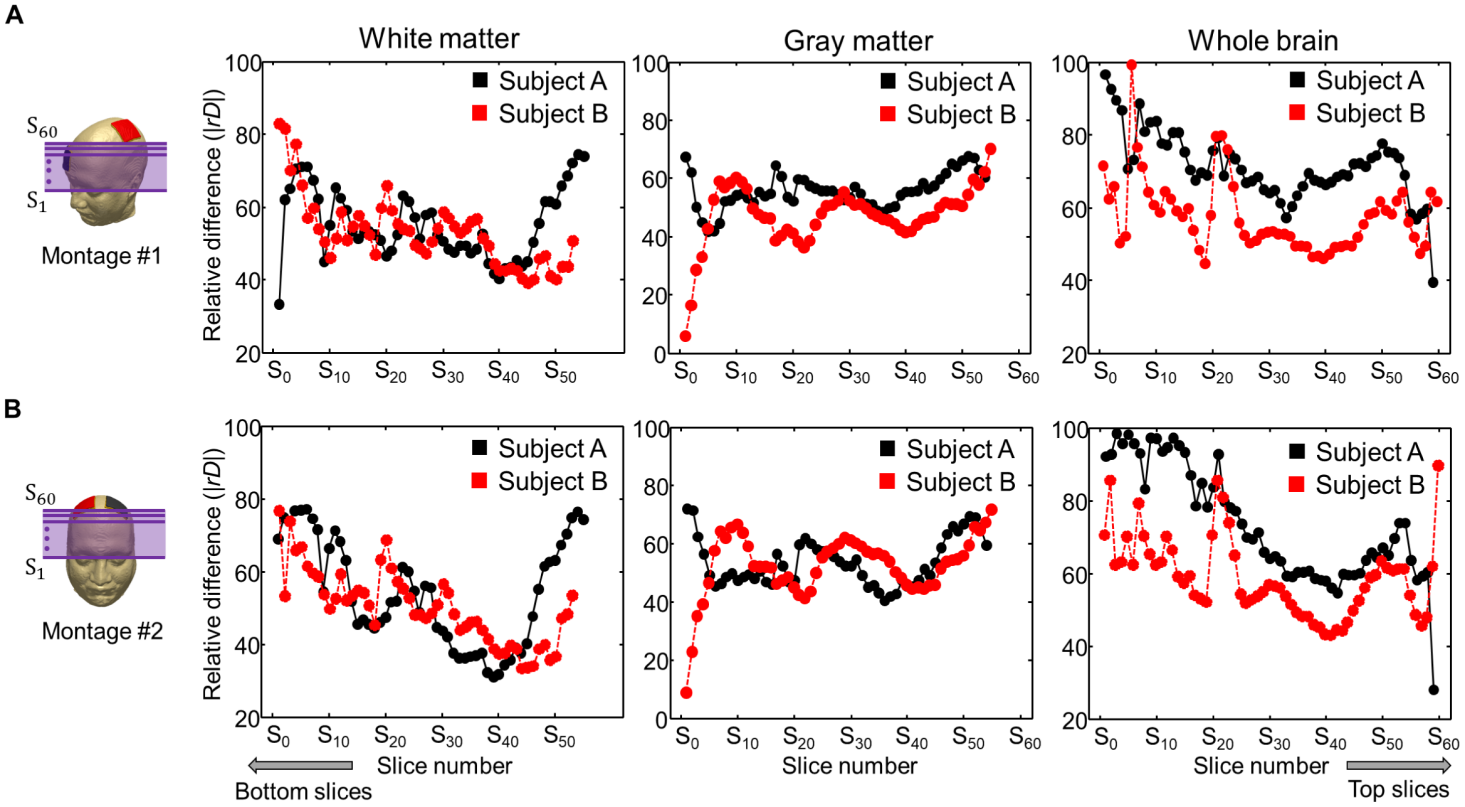
Comparison of relative difference (|*rD*|) between the current densities obtained from the two models. The current density in the brain tissues was calculated from the C3-FP2 **(A)** and F4-F3 **(B)** electrode montages.

Figure 6 represents comparisons of the current densities obtained from the two models by applying a relative difference (|*rD*|). The relative difference for two electrode montages was calculated in the white matter, gray matter, and entire brains of the two subjects. The differences ranged from 55 to 73% for the subject A and from 52 to 71% for the subject B. The minimum and maximum errors ranged from 15 to 98%.

### 3.4. Estimation of brain tissue response by different stimulation method

**Figure 7:**
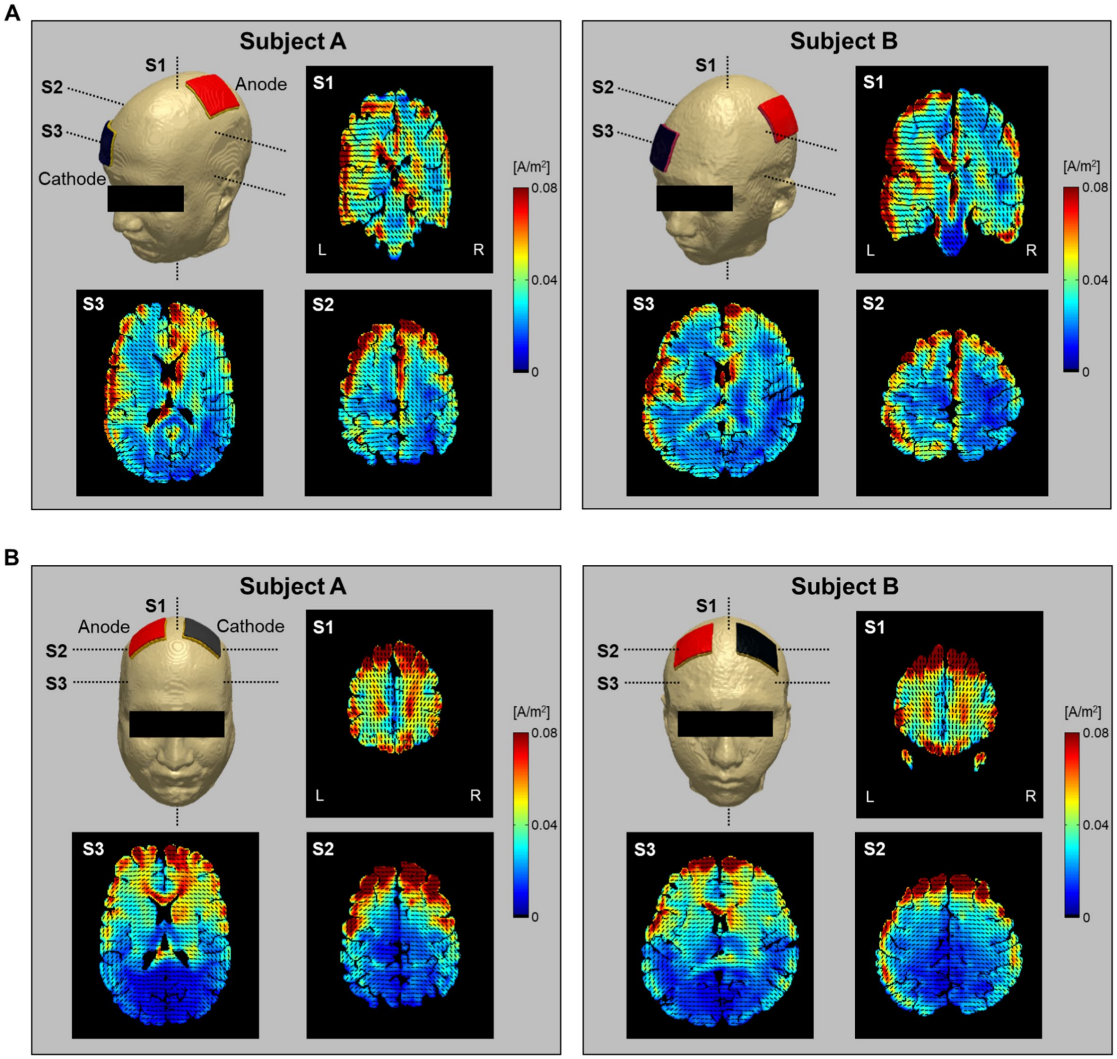
Estimation of current density distribution by electrical stimulation. The current density was calculated from the C3-FP2 **(A)** and F4-F3 **(B)** electrode montages using the conductivity tensor model. The current density distribution was imaged at single coronal slices of the heads near the anode electrode and at two axial slices of the head 75 and 25 mm apart from the skull. The vector field of current pathways is represented by black dashed lines.

Figure 7 shows the current density distribution in brains with two electrode montages (C3-FP2 and F4-F3). The current density images, which consist of one coronal and two axial slice positions, were calculated from the conductivity tensor model. From the C3-FP2 electrode montage in Figure 7A, the current density showed a high signal intensity which is consistent with the current flowing from the cortex where the anode electrode is located, through the deep brain region, and to the opposite cortex where the cathode electrode is located. In the F4-F3 electrode montage (Figure 7B), however, the current density was high in the cortex regions between the two electrodes but did not pass through the deep brain. Table 2 summarizes the measurements of current density in the entire brain. The C3-FP2 electrode montage showed higher signal values than the F4-F3 electrode montage in all slice positions.

**Table 2:** Measurements of the current density in two electrode montages.

Electrode montage	Subject A			Subject B		
	S1	S2	S3	S1	S2	S3
C3-FP2	0.043 ± 0.026	0.045 ± 0.034	0.040 ± 0.049	0.039 ± 0.026	0.052 ± 0.037	0.044 ± 0.031
F4-F3	0.036 ± 0.022	0.028 ± 0.017	0.019 ± 0.014	0.031 ± 0.020	0.030 ± 0.017	0.022 ± 0.019

Values were obtained from the entire brain tissues at three different slice positions.

## 4. Discussion

Computational modeling of electrical stimulation approaches, such as tDCS, still faces uncertainty regarding the optimal stimulation of specific anatomical structures in the brain that are affected by a given current stimulation. One reason is that there are no proper ways to measure the anisotropic conductivity distribution of each individual’s brains *in vivo*. In this study, we performed *in vivo* brain imaging using a conductivity tensor imaging (CTI) method from a 3 T MRI and obtained subject-specific anisotropic conductivity tensor images of two normal brains. The resulting conductivity tensors in the white matter, gray matter, and CSF regions were clearly distinguished and showed similar results to those reported in a previous study (Katoch et al., 2018). In particular, the conductivity tensor of white matter tends to exhibit position dependency and inter-subject variability depending on the degree of anisotropy. Therefore, compared to the isotropic conductivity model which is based on fixed values, regional differences could be observed in estimating the electric field and current density by using the conductivity tensor model.

At low frequencies below 10 kHz, externally injected currents are mostly blocked by cell membranes, and internal current flows are constrained within extracellular pathways. The white matter exhibits strong anisotropy because the myelinated fibers running in parallel along the fiber direction behave as electrical insulators at low frequencies. From the results of electrical stimulations shown in Figure 3A, the electric field calculated by the conductivity tensor model showed a focused distribution with higher signal intensity in the left frontoparietal lobe compared to the results from the isotropic conductivity model. In the frontal views, however, the electric field by the conductivity tensor model showed lower signal intensity in the supraorbital area. The overall signal intensity of the entire brain was high in both subjects as seen from the difference images between the two models in Figure 4A. Since the tissue response to electrical stimulation was higher in subject A than in subject B, the dynamic range of the electric field was different between two subjects.

The current density by the conductivity tensor model showed a focused distribution with high signal intensity along the current pathways. This high current density was observed in the white matter tissues such as corpus callosum, cingulum, and cortex regions around the electrodes. Especially, the high current density in the corpus callosum, which connects the left and right hemispheres, indicates that the anisotropy of the white matter is well reflected in the current density distribution produced by this model. The current density of gray matter showed a wider distribution than the white matter due to its higher isotropic conductivity. From the difference images in Figure 4B, the current density of the entire brain was high along the current pathway. This was also seen in the results of the current density streamlines in Figure 5. The current density streamlines of the isotropic conductivity model were widely distributed inside the brains whereas streamlines of the conductivity tensor model shows a more focused distribution between the two electrodes. Since the current densities are different between the two subjects, the dynamic ranges were different despite being performed under the same conditions. Therefore, our subject-specific conductivity tensor model may reflect the position dependency and inter-subject variability better than the isotropic model models.

For comparisons of the current densities between the two models, the relative difference (|*rD*|) analysis was applied to the brain tissues. Average relative difference in brain tissues were above 50% in both subjects. These large error values indicate that there is a significant difference between the two models. Similar errors were found when using white matter’s directional conductivity information based upon diffusion tensor information (Shahid et al., 2013; Rampersad et al., 2014; Opitz et al., 2015). Shahid et al. reported up to 48% higher strength of current density in the white matter compared to isotropic model. In these previous studies, however, anisotropic conductivity tensors were assumed to be proportional to water diffusion tensors with one fixed global scale factor.

To evaluate the brain response to electrical stimulation, we applied our subject-specific conductivity tensor models to image the current density distributions with two different electrode montages delivering tDCS (C3-FP2 and F4-F3). In Figure 7, the current density distributions at three different slice positions showed different patterns depending on the electrode montage as well as the individual brains. Specifically, the C3-FP2 montage in Figure 7A produced a more concentrated current density according to the current flow in subject A, but the amount of current density was larger in subject B. The current density showed higher signal intensity in the interhemispheric fissure and cortical regions around the two electrodes. The current density of the CSF region, which flows outside of the brain, was higher in subject B than in subject A. The overall signal intensity inside the brains was different between the two subjects in response to the injected currents. These results could be important for estimating the polarizing effects of individual brains by the electrical current (Purpura and McMurtry, 1965; Abascal et al., 2008). For the F4-F3 montage in Figure 7B, the current density was high in the cortical regions between the two electrodes in both subjects. However, the effect of CSF was more severe in subject B than in subject A.

It is important to determine how to evaluate tissue responses to electrical stimulation. As seen tissue responses may vary depending on individual brains and the stimulation location. Though the conductivity tensor information can be obtained using other imaging methods such as DT-MREIT, CTI has an advantage for tDCS treatment planning, since it does not require externally injecting currents into the head during MRI scans and treatments. The CTI-based head model exhibits potential in that it can consider position dependency and inter-subject variability. Although this study presented the results of only two normal brains and limited electrode positions, it showed a potential as an imaging tool for the evaluation of electrical stimulation approaches such as tDCS, through the accumulation of individual data from many samples and the subsequent implementation of statistical analysis.

The proposed CTI method has several limitations and requires careful considerations in its applications. First, the reconstructed images were geometrically registered, but the resolution of conductivity tensors imaging was limited. Moreover, there might be a partial volume effect that may result in field values being less accurate within the regional boundaries of each tissue type. Future studies can utilize higher resolution images to improve the head model (Marino et al., 2021). Second, since CTI uses MREPT and DWI data, a rigorous error analysis is desirable for propagating measurement noise and artifacts. Third, cell membranes are assumed to block low-frequency currents in CTI method. The CTI method in its current form could underestimate the low-frequency conductivity value of tissue, including cells with leaky membranes. To remedy this, it would be worthwhile to investigate a more sophisticated CTI model including such cells. A potential way to validate the CTI-based model is by combining surface voltage measurements using an EIT device or current density images from MRCDI (Abascal et al., 2008; Kasinadhuni et al., 2017). Future research focuses on optimizing targeted stimuli while exploring multiple electrode locations and recommending the optimal location based on the required amount of current for effective activation. This may be essential in the field of personalized tDCS treatment planning.

## 5. Conclusion

Feasibility of a personalized tDCS treatment planning was evaluated using a subject-specific head model with both conductivity tensors and anatomical information. The key ingredient is the CTI method that can produce anisotropic conductivity tensor images of the human brain using a 3 T clinical MRI without injecting external currents into the head. Once the subject-specific head model is constructed, various numerical simulations and analyses can be performed for different electrode configurations and current dosages. We suggest constructing subject-specific head models using the method described in this study and quantitatively analyzing internal distributions of the electric field and current density for tDCS treatment planning. Developing a software tool to automate the computational modeling process described in this study may facilitate the basic research and clinical applications of tDCS.

## Data availability statement

The original contributions presented in the study are included in the article/supplementary material, further inquiries can be directed to the corresponding authors.

## Ethics statement

The studies involving human participants were reviewed and approved by Institutional review board of Kyung Hee University (KHSIRB-18-073). The patients/participants provided their written informed consent to participate in this study.

## Author contributions

NK, YK, and SH: conceptualization. NK, BC, and TK: methodology. NK: software. YK and SS: validation. BC, NK, and SH: formal analysis. EY, SS, and YK: investigation. BC, TK, and JK: data curation. EY and JK: writing—original draft preparation. HK and JK: writing—review and editing. HK: visualization and supervision. All authors contributed to the finalization and approved the content of the manuscript.

## Funding

This work was funded by the National Research Foundation of Korea (NRF) grants funded by the Korea government (Nos. 2019R1A2C2088573, 2020R1I1A3065215, 2021R1A2C2004299, and 2022R1I1A1A01065565). This study was also supported by research fund from Chosun University, 2020.

## Conflict of interest

The authors declare that the research was conducted in the absence of any commercial or financial relationships that could be construed as a potential conflict of interest.

## Publisher’s note

All claims expressed in this article are solely those of the authors and do not necessarily represent those of their affiliated organizations, or those of the publisher, the editors and the reviewers. Any product that may be evaluated in this article, or claim that may be made by its manufacturer, is not guaranteed or endorsed by the publisher.

## Footnotes

^1^www.mrtrix.org

## References

[ref1] Abascal J. F. P. Arridge S. R. Atkinson D. Horesh R. Fabrizi L. De Lucia M.. (2008). Use of anisotropic modelling in electrical impedance tomography; description of method and preliminary assessment of utility in imaging brain function in the adult human head. NeuroImage 43, 258–268. doi: 10.1016/j.neuroimage.2008.07.023, PMID: 18694835

[ref2] Baumann S. B. Wozny D. R. Kelly S. K. Meno F. M. (1997). The electrical conductivity of human cerebrospinal uid at body temperature. I.E.E.E. Trans. Biomed. Eng. 44, 220–223. doi: 10.1109/10.554770, PMID: 9216137

[ref3] Bikson M. Rahman A. Datta A. (2012). Computational models of transcranial direct current stimulation. Clin. EEG Neurosci. 43, 176–183. doi: 10.1177/1550059412445138 22956646

[ref5] Chauhan M. Indahlastari A. Kasinadhuni A. K. Schar M. Mareci T. H. Sadleir R. J. (2018). Low-frequency conductivity tensor imaging of the human head in vivo using DT-MREIT: first study. IEEE Trans. Med. Imaging 37, 966–976. doi: 10.1109/TMI.2017.2783348, PMID: 29610075 PMC5963516

[ref6] Choi B. K. Katoch N. Park J. A. Kim J. W. Oh T. I. Kim H. J.. (2023). Measurement of extracellular volume fraction using magnetic resonance-based conductivity tensor imaging. Front. Physiol. 14:1132911. doi: 10.3389/fphys.2023.1132911, PMID: 36875031 PMC9983119

[ref7] Choi B. K. Katoch N. Park J. A. Ko I. O. Kim H. J. Kwon O. I.. (2020). Validation of conductivity tensor imaging using giant vesicle suspensions with different ion mobilities. Biomed. Eng. Online 19:35. doi: 10.1186/s12938-020-00780-5, PMID: 32448134 PMC7247266

[ref8] Datta A. Bansal V. Diaz J. Patel J. Reato D. Bikson M. (2009). Gyri-precise head model of transcranial direct current stimulation: improved spatial focality using a ring electrode versusconventional rectangular pad. Brain Stimul. 2, 201–207.e1. doi: 10.1016/j.brs.2009.03.005, PMID: 20648973 PMC2790295

[ref9] Datta A. Truong D. Minhas P. Parra L. C. Bikson M. (2012). Inter-individual variation during transcranial direct current stimulation and normalization of dose using mri-derived computational models. Front. Psych. 3:91. doi: 10.3389/fpsyt.2012.00091 PMC3477710 23097644

[ref10] Fregni F. Boggio P. S. Nitsche M. A. Rigonatti S. P. Pascual-Leone A. (2006). Cognitive effects of repeated sessions of transcranial direct current stimulation in patients with depression. Depress. Anxiety 23, 482–484. doi: 10.1002/da.20201, PMID: 16845648

[ref11] Gabriel C. Gabriel S. Corthout Y. E. (1996). The dielectric properties of biological tissues: I. literature survey. Phys. Med. Biol. 41, 2231–2249. doi: 10.1088/0031-9155/41/11/001, PMID: 8938024

[ref12] Gurler N. Ider Y. Z. (2017). Gradient-based electrical conductivity imaging using MR phase. Magn. Reson. Med. 77, 137–150. doi: 10.1002/mrm.26097, PMID: 26762771

[ref13] Huang Y. Dmochowski J. P. Su Y. Datta A. Rorden C. Parra L. C. (2013). Automated mri segmentation for individualized modeling of current flow in the human head. J. Neural Eng. 10:066004. doi: 10.1088/1741-2560/10/6/066004, PMID: 24099977 PMC3848963

[ref14] Jahng G. H. Lee M. B. Kim H. J. Woo E. J. Kwon O. I. (2021). Low-frequency dominant electrical conductivity imaging of in vivo human brain using high-frequency conductivity at Larmor-frequency and spherical mean diffusivity without external injection current. NeuroImage 225:117466. doi: 10.1016/j.neuroimage.2020.117466, PMID: 33075557

[ref15] Jeong W. C. Sajib S. Z. K. Katoch N. Kim H. J. Kwon O. I. Woo E. J. (2016). Anisotropic conductivity tensor imaging of in vivo canine brain using DT-MREIT. IEEE Trans. Med. Imaging 36, 124–131. doi: 10.1109/TMI.2016.2598546 28055828

[ref16] Kaden E. Kelm N. D. Carson R. P. Does M. D. Alexander D. C. (2016). Multi-compartment microscopic diffusion imaging. NeuroImage 139, 346–359. doi: 10.1016/j.neuroimage.2016.06.002, PMID: 27282476 PMC5517363

[ref17] Kasinadhuni A. Indahlastari A. Chauhan M. Schär M. Mareci T. Sadleir R. (2017). Imaging of current flow in the human head during transcranial electrical therapy. Brain Stimul. 10, 764–772. doi: 10.1016/j.brs.2017.04.125, PMID: 28457836 PMC5513732

[ref18] Katoch N. Choi B. K. Park J. A. Ko I. O. Kim H. J. (2021). Comparison of five conductivity tensor models and image reconstruction methods using MRI. Molecules 26:5499. doi: 10.3390/molecules26185499, PMID: 34576970 PMC8467711

[ref19] Katoch N. Choi B. K. Sajib S. Z. K. Lee E. A. Kim H. J. Kwon O. I.. (2018). Conductivity tensor imaging of *in vivo* human brain and experimental validation using giant vesicle suspension. IEEE Trans. Med. Imaging 38, 1569–1577. doi: 10.1109/TMI.2018.2884440 30507528

[ref20] Kwon O. I. Jeong W. C. Sajib S. Z. K. Kim H. J. Woo E. J. (2014). Anisotropic conductivity tensor imaging in MREIT using directional diffusion rate of water molecules. Phys. Med. Biol. 59, 2955–2974. doi: 10.1088/0031-9155/59/12/2955, PMID: 24841854

[ref21] Lee J. Bestmann S. Evans C. (2021). A future of current flow modelling for transcranial electrical stimulation? Curr. Behav. Neurosci. Rep. 8, 150–159. doi: 10.1007/s40473-021-00238-5

[ref22] Lindenmayer J. Kulsa M. K. C. Sultana T. Kaur A. Yang R. Ljuri I.. (2019). Transcranial direct-current stimulation in ultra-treatment-resistant schizophrenia. Brain Stimul. 12, 54–61. doi: 10.1016/j.brs.2018.10.002, PMID: 30316742

[ref23] Marino M. Cordero-Grande L. Mantini D. Ferrazzi G. (2021). Conductivity tensor imaging of the human brain using water mapping techniques. Front. Neurosci. 15:694645. doi: 10.3389/fnins.2021.694645, PMID: 34393709 PMC8363203

[ref24] Márquez-Ruiz J. Leal-Campanario R. Sánchez-Campusano R. Molaee-Ardekani B. Wendling F. Miranda P. C.. (2012). Transcranial direct-current stimulation modulates synaptic mechanisms involved in associative learning in behaving rabbits. Proc. Natl. Acad. Sci. U. S. A. 109, 6710–6715. doi: 10.1073/pnas.1121147109, PMID: 22493252 PMC3340065

[ref25] Miranda P. C. Lomarev M. Hallett M. (2006). Modeling thecurrent distribution during transcranial direct current stimulation. Clin. Neurophysiol. 117, 1623–1629. doi: 10.1016/j.clinph.2006.04.009 16762592

[ref26] Nitsche M. A. Paulus W. (2000). Excitability changes induced in the human motor cortex by weak transcranial direct current stimulation. J. Physiol. 527, 633–639. doi: 10.1111/j.1469-7793.2000.t01-1-00633.x, PMID: 10990547 PMC2270099

[ref27] Opitz A. Paulus W. Will S. Antunes A. Thielscher A. (2015). Determinants of the electric field during transcranial direct current stimulation. NeuroImage 109, 140–150. doi: 10.1016/j.neuroimage.2015.01.033 25613437

[ref28] Puonti O. Van Leemput K. Saturnino G. B. Siebner H. R. Madsen K. H. Thielscher A. (2020). Accurate and robust whole-head segmentation from magnetic resonance images for individualized head modeling. NeuroImage 219:117044. doi: 10.1016/j.neuroimage.2020.117044, PMID: 32534963 PMC8048089

[ref29] Purpura D. P. McMurtry J. G. (1965). Intracellular activities and evoked potential changes during polarization of motor cortex. J. Neurophysiol. 28, 166–185. doi: 10.1152/jn.1965.28.1.166, PMID: 14244793

[ref30] Rampersad S. M. Janssen A. M. Lucka F. Aydin Ü. Lanfer B. Lew S.. (2014). Simulating transcranial direct current stimulation with a detailed anisotropic human head model. IEEE Trans. Neural Syst. Rehabil. Eng. 22, 441–452. doi: 10.1109/TNSRE.2014.2308997, PMID: 24760939

[ref31] Reinhart R. M. Zhu J. Park S. Woodman G. F. (2015). Synchronizing theta oscillations with direct-current stimulation strengthens adaptive control in the human brain. Proc. Natl. Acad. Sci. U. S. A. 112, 9448–9453. doi: 10.1073/pnas.1504196112, PMID: 26124116 PMC4522782

[ref32] Sajib S. Z. K. Katoch N. Kim H. J. Kwon O. I. Woo E. J. (2017). Software toolbox for low-frequency conductivity and current density imaging using MRI. I.E.E.E. Trans. Biomed. Eng. 64, 2505–2514. doi: 10.1109/TBME.2017.2732502, PMID: 28767360

[ref33] Sajib S. Z. K. Kwon O. I. Kim H. J. Woo E. J. (2018). Electrodeless conductivity tensor imaging (CTI) using MRI: basic theory and animal experiments. Biomed. Eng. Lett. 8, 273–282. doi: 10.1007/s13534-018-0066-3, PMID: 30603211 PMC6208539

[ref34] Seo J. K. Woo E. J. (2012). Nonlinear inverse problems in imaging. Hoboken: John Wiley & Sons.

[ref35] Shahid S. S. Bikson M. Salman H. Wen P. Ahfock T. (2014). The value and cost of complexity in predictive modelling: role of tissue anisotropic conductivity and fibre tracts in neuromodulation. J. Neural Eng. 11:036002. doi: 10.1088/1741-2560/11/3/036002, PMID: 24737098

[ref36] Shahid S. Wen P. Ahfock T. (2013). Numerical investigation of white matter anisotropic conductivity in defining current distribution under tDCS. Comput. Methods Prog. Biomed. 109, 48–64. doi: 10.1016/j.cmpb.2012.09.001, PMID: 23040278

[ref37] Smith S. M. Jenkinson M. Woolrich M. W. Beckmann C. F. Behrens T. E. Johansen-Berg H.. (2004). Advances in functional and structural MR image analysis and implementation as FSL. NeuroImage 23, S208–S219. doi: 10.1016/j.neuroimage.2004.07.051, PMID: 15501092

[ref38] Stagg C. J. Nitsche M. A. (2011). Physiological basis of transcranial direct current stimulation. Neuroscientist 17, 37–53. doi: 10.1177/1073858410386614 21343407

[ref39] Tournier J. D. Smith R. Raffelt D. Tabbara R. Dhollander T. Pietsch M.. (2019). MRtrix3: a fast, flexible and open software framework for medical image processing and visualisation. NeuroImage 202:116137. doi: 10.1016/j.neuroimage.2019.116137, PMID: 31473352

[ref40] Tuch D. S. Wedeen V. J. Dale A. M. George J. S. Belliveau J. W. (2001). Conductivity tensor mapping of the human brain using diffusion tensor MRI. Proc. Natl. Acad. Sci. 98, 11697–11701. doi: 10.1073/pnas.171473898, PMID: 11573005 PMC58792

[ref41] Woods A. J. Antal A. Bikson M. Boggio P. S. Brunoni A. R. Celnik P.. (2016). A technical guide to tDCS, and related non-invasive brain stimulation tools. Clin. Neurophysiol. 127, 1031–1048. doi: 10.1016/j.clinph.2015.11.012, PMID: 26652115 PMC4747791

